# Nocardiosis pulmonar y del sistema nervioso central: el alcoholismo como factor de inmunocompromiso

**DOI:** 10.7705/biomedica.6606

**Published:** 2023-03-30

**Authors:** Adriana Isabel Márquez, Eduardo Mora, Andrés Felipe Bernal, Andrés Felipe Salazar, Diana Patricia Mora, Ledmar Jovanny Vargas

**Affiliations:** 1 Servicio de Infectología, Hospital Universitario San Rafael, Tunja, Colombia Hospital Universitario San Rafael Tunja Colombia; 2 Servicio de Radiología, Hospital Universitario San Rafael, Tunja, Colombia Hospital Universitario San Rafael Tunja Colombia; 3 Hospital Universitario San Rafael, Tunja, Colombia Hospital Universitario San Rafael Tunja Colombia; 4 Servicio de Laboratorio Clínico, Hospital Universitario San Rafael, Tunja, Colombia Hospital Universitario San Rafael Tunja Colombia; 5 Servicio de Epidemiología, Hospital Universitario San Rafael, Tunja, Colombia Hospital Universitario San Rafael Tunja Colombia

**Keywords:** Nocardia, nocardiosis, absceso encefálico, alcoholismo, inmunocompetencia, hospedero inmunocomprometido, Nocardia, Nocardia infections, brain abscess, alcoholism, immunocompetence, immunocompromised host

## Abstract

La nocardiosis es una enfermedad de distribución mundial; de forma habitual se encuentra en zonas tropicales y afecta principalmente a pacientes inmunocomprometidos, sin embargo, también existen casos reportados de infección en personas inmunocompetentes. Esta infección es causada por actinomicetos del género *Nocardia* spp. que son bacterias Gram positivas, saprófitos ambientales. Aunque la exposición a *Nocardia* spp. es casi universal, solo una pequeña fracción de las personas expuestas desarrollan la enfermedad.

Se presenta el caso de un hombre de 47 años, sin dato de inmunosupresión, procedente de un área rural de Boyacá, que consultó por un cuadro clínico de cefalea intensa e intermitente, con parestesias y, finalmente, alteración del estado de conciencia. Se practicó una resonancia magnética cerebral, en la que se evidenció una lesión que ocupaba espacio de localización córtico-subcortical en la región fronto-témporo-parietal izquierda, con efecto compresivo y desplazamiento de las cavidades del sistema ventricular. Se sospechó, inicialmente, una lesión neoplásica o un absceso cerebral.

El paciente fue sometido a una resección quirúrgica, y el cultivo de la lesión documentó *Nocardia africana/nova*; en estudios posteriores, se evidenció un posible foco pulmonar primario. Como único factor de riesgo en el paciente, se documentó alcoholismo. Completó seis semanas de tratamiento antibiótico intrahospitalario con evolución clínica y radiológica, y egresó con plan de un año de terapia antibiótica ambulatoria. Aunque la enfermedad por *Nocardia* spp. afecta principalmente a pacientes inmunocomprometidos, la “evidencia” clínica demuestra que este microorganismo también puede ser una amenaza para individuos sin los factores de riesgo tradicionales para inmunosupresión.

## Abstract (en)

Nocardiosis is a disease with worldwide distribution. It is usually found in tropical areas and mainly affects immunocompromised patients, however, there are also cases where its infection has been reported in immunocompetent patients. This pathology is caused by bacteria known as *Nocardia* spp., which are gram-positive microorganisms and environmental saprophytes, and although exposure to *Nocardia* spp. is almost universal, only a small fraction of exposed people develops the disease.

We present the case of a 47-year-old man, with no evidence of immunosuppression, from a rural area of Boyacá, who was admitted due to intense and intermittent headache accompanied by paresthesia and, finally, a decrease in consciousness. A brain magnetic resonance was performed and evidenced a fronto-temporo- occipital space-occupying lesion in the cortico-subcortical region with a compressive effect and displacement of the ventricular system cavities. It was suspected at first a neoplastic lesion or a brain abscess. The lesion was surgically resected, and its culture showed *Nocardia africana/nova*. In later studies a possible primary pulmonary focus was evidenced. Alcoholism was the only risk factor documented. The patient completed 6 weeks of hospital antibiotic treatment with favorable clinical and radiological evolution and was discharged with a 1-year plan of outpatient antibiotic therapy. Although *Nocardia* spp. mainly affects immunocompromised patients, evidence shows that this microorganism can also be a threat to individuals without traditional immunosuppression risk factors.

La nocardiosis es una rara infección oportunista que compromete principalmente a pacientes con alteraciones de la inmunidad celular, como aquellos con síndrome de inmunodeficiencia adquirida o con trasplante de órganos. Esta infección es causada por bacterias de un género de actinomicetos conocido como *Nocardia* spp. ^1^^,^^2^. Son microorganismos Gram positivos, saprófitos ambientales y, aunque la exposición a *Nocardia* spp. es frecuente en el ambiente, solamente una pequeña fracción de personas expuestas desarrollan la enfermedad ^3^^,^^4^. Los factores de riesgo incluyen profundas deficiencias en la inmunidad celular, principalmente relacionadas con trasplantes de órgano sólido o hematopoyético, uso de esteroides, neoplasias malignas o infección por el virus de la inmunodeficiencia humana ^5^^,^^6^.

Clínicamente, puede presentarse como una enfermedad cutánea cuando la bacteria se inocula en la piel o, pulmonar, cuando la bacteria se inhala y llega a los pulmones; además, puede diseminarse desde estos focos iniciales a otros órganos ^4^^,^^6^. El objetivo de este informe es presentar el caso de un paciente con nocardiosis del sistema nervioso central y pulmonar, sin documentación de inmunosupresión.

## Caso clínico

Se trata de un hombre de 47 años, procedente de un área rural de Boyacá. Fue hospitalizado por presentar un cuadro clínico de 20 días de evolución de cefalea intermitente, intensa, junto con parestesias y disminución de la fuerza en la mano derecha; además, un episodio convulsivo tónico-clónico generalizado de cerca de 10 minutos de duración y con periodo postictal de 20 minutos de duración. Dos días antes de su hospitalización presentó somnolencia, la cual progresó hasta estupor en el día de su ingreso. Sufría de hipertensión arterial sistémica en tratamiento con losartán y, como antecedente importante, reportó consumo de alcohol todos los días -con frecuencia hasta la embriaguez- desde la adolescencia.

Al ingreso, se encontraba en mal estado general, con importante compromiso neurológico, en estupor, con 5/15 en la escala de Glasgow (sin respuesta ocular ni verbal, ni respuesta motora al dolor), pupilas hiporreactivas e isocóricas de 3 ml y hemiparesia derecha.

**Figura 1:**
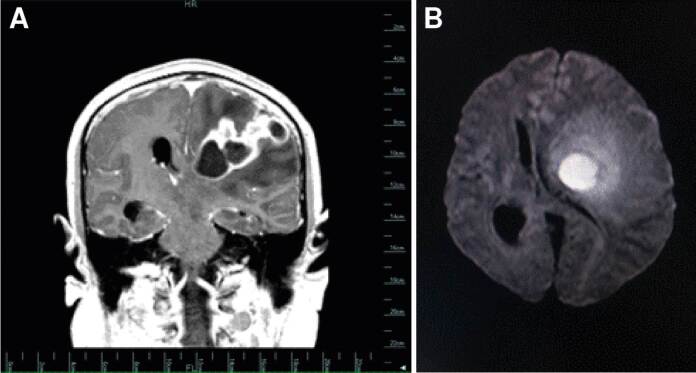
Resonancia magnética cerebral con equipo de 1.5 Tesla con protocolo rutinario para cerebro, sin administración endovenosa de contraste paramagnético y con contraste, con secuencia de difusión y mapa paramétrico (mapa del coeficiente de difusión aparente). A. Secuencia potenciada en T1 con contraste paramagnético: se observa una lesión cortico- subcortical que ocupa espacio, en la región fronto-témporo-parietal izquierda, que compromete la sustancia blanca profunda y el ganglio basal superior; existe un componente sólido que se realza intensamente y áreas de caracterización quística con realce periférico en patrón anular. B. En la secuencia potenciada en difusión (*Diffusion-weighted imaging*), tiene comportamiento de patrón restrictivo, efecto compresivo y desplazamiento de las cavidades del sistema ventricular con herniación del cíngulo por debajo de la hoz del cerebro.

En la resonancia magnética cerebral se demostró una lesión intraaxial que ocupaba espacio, multiquística, compleja y de localización córtico-subcortical, en la la región fronto-temporo-parietal del hemisferio cerebral izquierdo, que comprometía la sustancia blanca profunda y el ganglio basal superior, con efecto compresivo y desplazamiento de los ventrículos ipsilaterales y herniación del cíngulo por debajo de la hoz del cerebro, característica de neoplasia o infección (figura 1).

Ante estos resultados, se decidió practicar una craneotomía descompresiva de urgencia con resección de la lesión supratentorial, cuyo hallazgo intraoperatorio fue el de un tumor abscedado.

**Figura 2:**
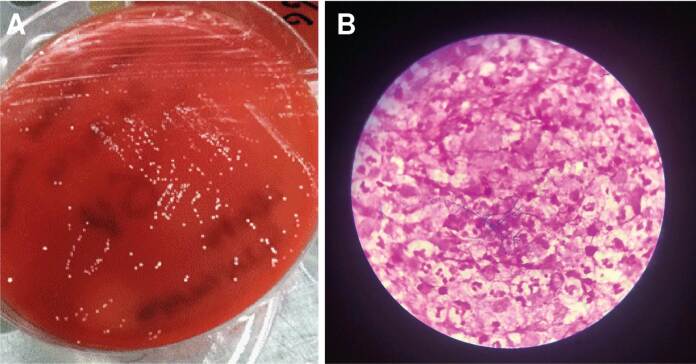
A. Colonias de *Nocardia africana/nova* en agar sangre. Muestra: absceso cerebral. B. Se observan estructuras bacterianas filamentosas Gram positivas. Tinción de Gram, 100X.

Se tomó muestra de la lesión y la muestra fue enriquecida en caldo tioglicolato, se cultivó en agar sangre y agar chocolate, y se incubó a 35-37 °C por cuatro días en aerobiosis. Se obtuvo crecimiento de colonias blancas rugosas y quebradizas. En la tinción de Gram se observaron estructuras bacterianas Gram positivas filamentosas (figura 2); la tinción de Ziehl-Neelsen fue negativa. Se identificaron el género y la especie con la técnica de MALDI-TOF, y se documentó *Nocardia africana/nova*. El estudio de histopatología descartó una lesión tumoral.

**Figura 3:**
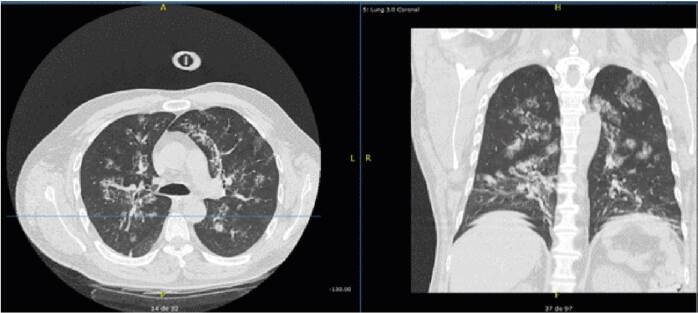
Cortes axial y coronal que evidencian múltiples opacidades con áreas de atenuación en vidrio esmerilado de predominio central, con compromiso de ambos campos pulmonares, que sugiere ser el foco primario de la diseminación de la infección por *Nocardia* spp.

Además, como parte de los estudios de extensión, se ordenó tomografía computarizada de tórax, la que documentó áreas con aumento de la atenuación en vidrio esmerilado distribuidas en forma parchada en ambos campos pulmonares, con predominio central (figura 3). Se hicieron lavado broncoalveolar y cultivo. No se obtuvo aislamiento de *Nocardia* spp. La prueba molecular de identificación de *Mycobacterium tuberculosis* y el cultivo para micobacterias fueron negativos. Asimismo, la prueba rápida de HIV fue negativa y el recuento de linfocitos T CD3, CD4 y CD8 no demostró ninguna alteración.

El paciente fue tratado con trimetoprim-sulfametoxazol a dosis altas (15 mg/kg/día) más linezolid intravenoso, 600 mg cada 12 horas. Después de dos semanas de tratamiento, presentó compromiso hematológico con sospecha de mielosupresión en relación con el linezolid; por esta razón, se cambió el tratamiento a imipenem cilastatina y se continuó con el trimetoprim-sulfametoxazol a una dosis menor de 10 mg/kg/día. Se practicó un segundo procedimiento de drenaje de la colección y los nuevos estudios microbiológicos resultaron negativos. Luego de completar seis semanas de tratamiento intrahospitalario, con evolución clínica y radiológica favorable, se dio egreso para continuar tratamiento ambulatorio con trimetoprim-sulfametoxazol y claritromicina, en un plan de un año. El paciente egresó con secuelas motoras de la hemiparesia derecha, con mejoría con el proceso de rehabilitación documentado en el seguimiento clínico.

## Discusión

El género *Nocardia* incluye más de 90 especies, muchas de las cuales han demostrado ser patógenas para los humanos ^1^^,^^7^. Inicialmente, la clasificación se basó en las propiedades bioquímicas, pero posteriormente, se hicieron modificaciones basadas en técnicas moleculares y muchos aislamientos correspondientes al complejo N. asteroides fueron reclasificados ^1^. *Nocardia africana* se identificó en el 2001 en muestras pulmonares, pertenece al complejo *Nocardia nova* (*N. nova, N. elegans, N. veterana, N. kruczakiae y N. africana*) ^1^. *Nocardia farcinica* es la especie descrita con mayor frecuencia en las infecciones pulmonares y extrapulmonares, y *N. nova complex* se identifica como agente patógeno, especialmente en pacientes con trasplantes de órgano o con uso de esteroides; los casos en inmunocompetentes son esporádicos ^1^^,^^8^^,^^9^.

Las vías respiratorias son la principal puerta de entrada de *Nocardia* spp.; como consecuencia, alrededor del 50 al 70 % de los pacientes con nocardiosis tienen afectación pulmonar ^10^. La nocardiosis pulmonar y extrapulmonar comúnmente afecta a pacientes debilitados con condiciones predisponentes, especialmente a inmunodeprimidos debido a trasplante de órganos, uso de corticosteroides, neoplasias malignas o HIV ^3^^,^^8^^,^^9^^,^^11^. Con menor frecuencia, se ha encontrado alguna relación con diabetes mellitus, alcoholismo, enfermedad granulomatosa crónica, proteinosis alveolar y enfermedad pulmonar estructural ^5^^,^^6^^,^^11^.

El alcohol es una de las sustancias de uso más común en la población humana ^12^; sus propiedades adictivas pueden resultar en un consumo crónico y excesivo del mismo. Los efectos del alcohol sobre el sistema inmunitario no están del todo claros: el consumo crónico de alcohol tiene efectos tanto en la reacción inmunológica innata como en la adaptativa. En cuanto a las infecciones bacterianas, la mayor relación se ha encontrado con infecciones pulmonares. Se cree que el alcohol produce cambios en el perfil de citocinas y en los niveles de especies reactivas de oxígeno, favoreciendo el estrés oxidativo y la disfunción de los macrófagos alveolares; asimismo, el reclutamiento y la función de los neutrófilos están alterados, lo cual genera mayor daño tisular en los alvéolos pulmonares ^12^^-^^14^. En cuanto a nocardiosis y alcoholismo, si bien los datos son escasos, Martínez, *et al*., reportaron el alcoholismo como principal factor relacionado con la diseminación al sistema nervioso central desde un foco un pulmonar de *Nocardia* spp. Considerando la importancia de la actividad de neutrófilos y macrófagos, y la activación de la respuesta inmunológica mediada por células T, el impacto en la disfunción inmunológica relacionada con el alcoholismo podría contribuir a la diseminación de la infección.

En Colombia, el consumo de alcohol hace parte de la cultura de muchos territorios. Boyacá es la región con mayor consumo de alcohol; el 92 % de la población ha consumido alguna vez alcohol en la vida, valor mucho mayor del promedio nacional, que se sitúa en el 84 %. La edad más frecuente de inicio de consumo son los 18 años ^15^. Estos datos deben alertar sobre los efectos del alcoholismo en la población de la región, incluyendo infecciones oportunistas que con poca frecuencia son consideradas.

En Colombia, se han reportado algunos casos de nocardiosis diseminada y con compromiso del sistema nervioso central en individuos sin los clásicos factores de riesgo. Se han caracterizado por ser pacientes que han consultado al servicio médico en repetidas ocasiones y han recibido manejo empírico para procesos infecciosos más comunes, antes del diagnóstico de *Nocardia* spp. Lo anteriormente mencionado evidencia el reto clínico que es el diagnóstico de esta condición, especialmente, en población sin inmunosupresión conocida; en ninguno de los casos se documentó alcoholismo como factor predisponente ^10^^,^^16^^,^^17^.

En cuanto al tratamiento, el trimetoprim-sulfametoxazol ha sido la base del esquema antimicrobiano; sin embargo, dado el aumento de resistencia principalmente en *N. farcinica*, se recomienda confirmar su sensibilidad ^3^. En caso de enfermedad grave -como el compromiso del sistema nervioso central- la terapia debe ser combinada; el linezolid aparece como uno de los medicamentos con mayor actividad contra *Nocardia* spp. y una buena opción de combinación, pero con frecuencia se ve limitada por su potencial mielosupresión en el uso crónico. Otras alternativas, dependiendo de la especie de *Nocardia* spp., son el clavulanato de amoxicilina, la ceftriaxona, el imipenem, la amikacina, la claritromicina, la tigeciclina y la moxifloxacina. Nova complex se caracteriza por su resistencia al clavulanato de amoxicilina, la moxifloxacina y, con frecuencia, a la cefriaxona ^3^^,^^18^. La duración del tratamiento en caso de compromiso del sistema nervioso central debe ser, al menos, de un año ^19^. La cirugía es con frecuencia parte del manejo para la nocardiosis cerebral. En algunos casos, se realiza el procedimiento por sospecha de lesión tumoral o absceso cerebral de otra etiología; en otras ocasiones, como control local del proceso infeccioso.

Aunque la nocardiosis no es frecuente en nuestro medio, es importante para el médico su reconocimiento y la sospecha como diagnóstico de exclusión frente a otras causas de absceso cerebral, más aún, cuando se presenta también compromiso pulmonar. La nocardiosis es un diagnóstico diferencial de los abscesos cerebrales en pacientes con inmunosupresión clásica, como individuos con trasplantes, en tratamiento con esteroides o con HIV. Sin embargo, dada la complejidad de la reacción inmunitaria a estos microrganismos, debemos considerar factores con frecuencia olvidados, como el alcoholismo.

## Conclusiones

Aunque la enfermedad por *Nocardia* spp. afecta principalmente a pacientes inmunocomprometidos, la evidencia muestra que este microorganismo también puede ser una amenaza para individuos aparentemente sanos o con condiciones adquiridas, como el alcoholismo.

Es importante buscar el diagnóstico etiológico de los abscesos cerebrales y, en caso de *Nocardia* spp., identificar la especie e, idealmente, determinar la sensibilidad a los antimicrobianos para decidir la mejor opción terapéutica para el paciente.
